# Thermal Decomposition of Core–Shell-Structured RDX@AlH_3_, HMX@AlH_3_, and CL-20@AlH_3_ Nanoparticles: Reactive Molecular Dynamics Simulations

**DOI:** 10.3390/nano14221859

**Published:** 2024-11-20

**Authors:** Zijian Sun, Lei Yang, Hui Li, Mengyun Mei, Lixin Ye, Jiake Fan, Weihua Zhu

**Affiliations:** Institute for Computation in Molecular and Materials Science, School of Chemistry and Chemical Engineering, Nanjing University of Science and Technology, Nanjing 210094, China; zijian.sun@njust.edu.cn (Z.S.); yanglei21@njust.edu.cn (L.Y.); huili1995@njust.edu.cn (H.L.); 3115857405@njust.edu.cn (M.M.); lixin715@njust.edu.cn (L.Y.); fan_jk@njust.edu.cn (J.F.)

**Keywords:** reactive molecular dynamics, core–shell-structured aluminized hydride explosive, morphology evolution, decomposition kinetics, aluminized clusters

## Abstract

The reactive molecular dynamics method was employed to examine the thermal decomposition process of aluminized hydride (AlH_3_) containing explosive nanoparticles with a core–shell structure under high temperature. The core was composed of the explosives RDX, HMX, and CL-20, while the shell was composed of AlH_3_. It was demonstrated that the CL-20@AlH_3_ NPs decomposed at a faster rate than the other NPs, and elevated temperatures could accelerate the initial decomposition of the explosive molecules. The incorporation of aluminized hydride shells did not change the initial decomposition mechanism of the three explosives. The yields of the main products (NO, NO_2_, N_2_, H_2_O, H_2_, and CO_2_) were investigated. There was a large number of solid aluminized clusters produced during the decomposition, mainly Al_m_O_n_ and Al_m_C_n_ clusters, together with Al_m_N_n_ clusters dispersed in the Al_m_O_n_ clusters.

## 1. Introduction

Energetic materials are a class of materials that possess high energy densities and specialized chemical properties, with applications in the fields of military, civilian demolition, aerospace, and industrial manufacturing [1,2]. Among them, 1,3,5-trinitro-1,3,5-triazinane (RDX), octahydro-1,3,5,7-tetranitro-1,3,5,7-tetrazocine (HMX), and hexanitrohexaazaisowurtzitane (CL-20) are commonly used high explosives. RDX is widely used in artillery shells, landmines, and other military munitions due to its high energy density and stability [3,4]. HMX has an even higher energy density than RDX and is commonly used for military applications requiring large amounts of energy, such as nuclear weapon fuses and missile systems [4,5]. CL-20 belongs to a new generation of high explosives with an exceptionally high energy density [6], which makes it one of the most powerful explosives known today. It can be utilized in sophisticated weaponry, including hypersonic weapons and missiles [7].

Hydrogen energy offers a significant opportunity for the development of a clean energy source, offering a promising pathway towards a green and low-carbon transition. Aluminum hydride (AlH_3_) is identified as one of the most promising materials for hydrogen storage [8,9,10,11]. In the aerospace industry, AlH_3_ exhibits a number of advantageous properties, including high hydrogen content, high heat of combustion, low molecular weight of the combustion products, and relatively high thermal decomposition temperature. Additionally, it is an environmentally friendly material. Partially replacing aluminum powder with AlH_3_ has been demonstrated to markedly enhance the energy density of solid propellants, rendering them an optimal high-energy fuel. Thermochemical calculations demonstrated that AlH_3_ generates lower flame temperatures and higher specific impulses in comparison to aluminum fuels [12]. DeLuca et al. [13] conducted an experiment to assess the chemical and physical properties of AlH_3_-based composite solid propellants in addition to their ballistic characteristics. A comparison of the flame structures of AlH_3_ and aluminum-plated propellants revealed that the performance of the AlH_3_-based propellants was superior to that of the Al-based propellants. The ignition and combustion behaviors of aluminum hydride (α-AlH_3_) investigated by Yong et al. [14] demonstrated that both the ignition temperature and the temperature at which hydrogen is released increase with the increase in the heating rate. In addition, as the oxygen concentration increases, the combustion time and intensity decrease. Bazyn et al. [15] conducted a comparative analysis of the high-pressure combustion characteristics of aluminum hydride and aluminum in carbon dioxide and oxygen. The findings demonstrate that hydrogen is desorbed at a temperature significantly below the ignition temperature threshold of aluminum, and the released hydrogen is subsequently oxidized alongside the decomposition products of the oxidant. Additionally, the temperatures of the gas-phase and solid-state products were found to be highly comparable for both components. These investigations suggest that aluminum hydride possesses considerable potential for enhancing the explosive properties of energetic materials.

The molecular dynamics (MD) method is an effective approach for simulating the decomposition processes of energetic materials at the atomic level due to their complex chemical behaviors and extremely high risk. The reactive MD (RMD) method extends the time scale of the simulation and the size of the simulated system, making it a promising theoretical approach for exploring the pyrolysis and combustion reactions of energetic materials. Recently, the RMD has been successfully employed to simulate the thermal decomposition reactions of explosives and fuels [16,17,18,19,20,21,22,23]. Mei et al. [24] conducted molecular dynamics simulations of the thermal decomposition processes of nano-AlH_3_/TNT and nano-AlH_3_/CL-20 composites, and the findings indicate that nano-AlH_3_ particles facilitate the decomposition of explosives molecules. The findings by Ji et al. [25] indicate that the decomposition of HMX molecules in the Al/HMX composite is accelerated by the addition of Al. Subsequently, Ji et al. [26] conducted RMD simulations of the combustion process of aluminum-containing explosives in an oxygen environment. The results indicate that oxygen is seldom directly involved in the initial decomposition of explosive molecules, but instead primarily reacts with intermediates and free radicals. Additionally, the combustion process was observed to produce a significant number of solid aluminized clusters. The aforementioned studies demonstrate that the RMD can successfully simulate the thermal decomposition of aluminum- or aluminum hydride-containing explosives with a large size.

In this work, nanoparticles (NPs) of the explosives RDX, HMX, and CL-20 were used as cores to be assembled with shells of AlH_3_ to build three core–shell-structured composites named RDX@AlH_3_ NP, HMX@AlH_3_ NP, and CL-20@AlH_3_ NP, respectively. Then, their thermal decomposition processes at different temperatures were investigated using the RMD method. Our main objective was to study the impact of temperature on the thermal decomposition of RDX@AlH_3_, HMX@AlH_3_, and CL-20@AlH_3_ NPs and to examine the differences in their decomposition mechanisms.

## 2. Computational Methods

**Figure 1:**
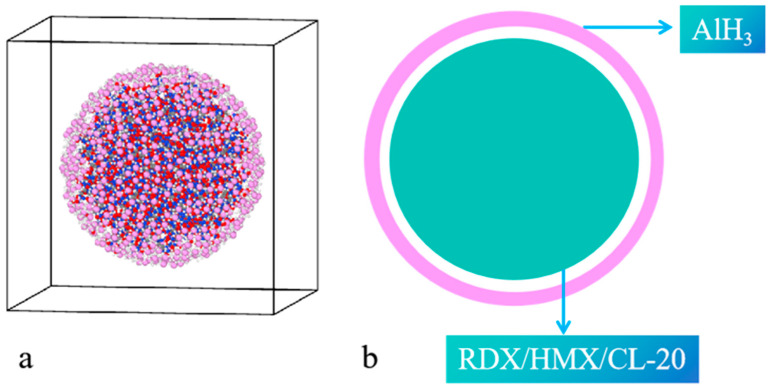
(**a**) Models of RDX/HMX/CL-20@AlH_3_ NPs, (**b**) schematic diagram of aluminized hydride explosives with a core–shell structure. C, H, O, N, and Al atoms are represented by gray, white, red, blue, and pink balls, respectively.

All RMD simulations were performed utilizing the ReaxFF reactive force field, implemented within the LAMMPS software package (Version: 5 Jun 2019) [27]. The reactive force field uses bond-order formalism and polarizable charges to accurately describe reactive and non-reactive interactions. ReaxFF has been employed extensively for the high-temperature pyrolysis of energetic materials. The ReaxFF parameters developed by Mai et al. [24] have been successfully utilized to simulate the pyrolysis and combustion of aluminized and hydrogenated aluminum explosives. The unit cell structures of RDX, HMX, and CL-20 were derived from experimental data [28,29,30]. We firstly constructed aluminum shells with a diameter of 6 nm and a thickness of 0.1 nm. Then, nanoparticles of RDX with a diameter of 5 nm were cut from the unit cell of RDX. Subsequently, the aluminum shells and the nanoparticles were assembled together, as illustrated in Figure 1. Finally, the initial RDX@AlH_3_ NPs were put at the center of the simulation box. It was demonstrated that the impact of periodicity on the simulation outcomes can be eliminated by setting the side length of the simulation box to be approximately twice the diameter of the nanoparticles [31]. Consequently, the side length of the box was set to 8 nm in the present simulations. The number of molecules and atoms included in the constructed models is listed in Table 1.

As an illustrative example, the RDX@AlH_3_ NPs were subjected to a 10 ps relaxation process utilizing the isothermal–isobaric ensemble (NPT) (T = 298 K, P = 1 atm) until the internal stress reached zero, ultimately yielding a stable structure. The relaxation process was conducted using an Andersen thermostat and a Nosé–Hoover thermostat to regulate the system pressure and temperature, respectively. The relaxed structure obtained was then employed as an input model for the subsequent simulations. The canonical ensemble (NVT) was used to simulate the decomposition of the RDX@AlH_3_ model, with the system temperature regulated by the Nosé–Hoover thermostat. Four temperatures were selected for the simulated systems: 2100, 2400, 2700, and 3000 K. The total duration of the simulation was set to 300 ps with a time step of 0.1 fs, and the damping constant was set to 100 fs. The construction and simulation processes of HMX@AlH_3_ and CL-20@AlH_3_ NPs were similar to those of RDX@AlH_3_ NPs.

## 3. Results and Discussion

### 3.1. Morphological Evolution

**Figure 2:**
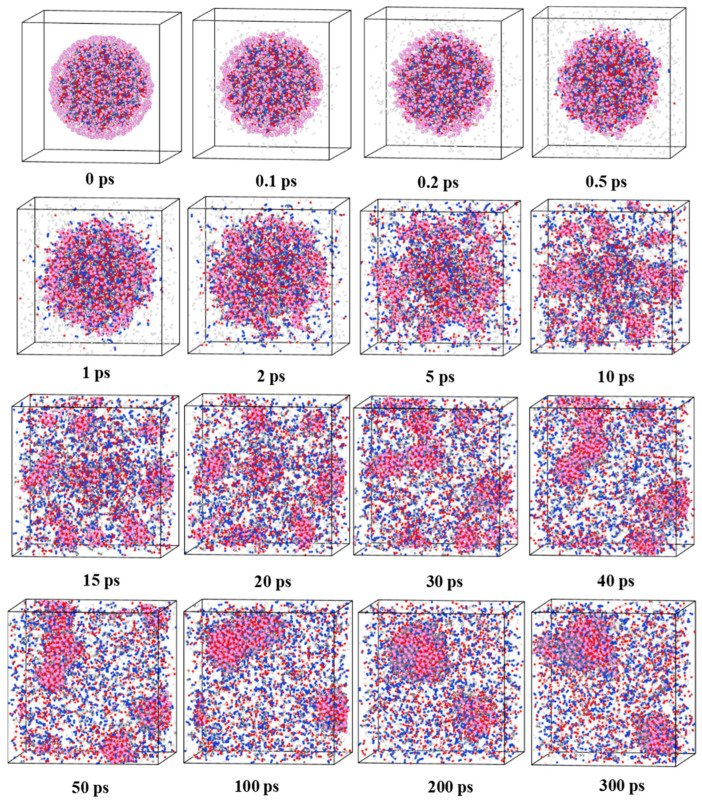
Snapshots of the morphology of RDX@AlH_3_ NPs at 2400 K.

In order to gain a comprehensive understanding of the thermal decomposition process of the three AlH_3_-containing explosive NPs, their morphological evolution was examined. AlH_3_ is susceptible to decompose to produce metallic aluminum and hydrogen gas when subjected to an increasing temperature, resulting in a decrease in its thermal stability. Nevertheless, nanocrystalline AlH_3_ displays greater thermal sensitivity [32]. Figure 2 depicts the decomposition process of RDX@AlH_3_ NPs at 2400 K. As illustrated in Figure 2, the release of hydrogen gas commenced at 0.1 ps. Song et al. [12] reported that the hydrogen bubbles in the shell have a high kinetic energy and are able to break through the thin layer. As the temperature rises, the thrust of the hydrogen bubbles increases, resulting in the ejection of a great number of aluminum clusters from the nanoparticles. This finding is in accordance with the results observed in our simulation. During the initial reaction stage from 0 to 2 ps, the RDX@AlH_3_ NPs gradually expanded, accompanied by the production of a considerable quantity of hydrogen gas and free radicals within the system. As the reaction time increased from 2 to 15 ps, the decomposition rate of RDX accelerated. The thrust generated by the substantial quantity of gas produced in the RDX@AlH_3_ NPs then facilitated the splitting of the aluminum shell, thereby promoting the formation of aluminum clusters. Between 15 and 40 ps, as the reaction progressed, the RDX molecules underwent complete decomposition, the particles expanded significantly, and the system was filled with gaseous products and free radicals. As a consequence of thermal motion, the oxide clusters collided with one another at 40 ps.

In the subsequent reactions, the number of the oxide clusters decreased, while the number of atoms contained in each cluster increased. Upon the completion of the pyrolysis process, the aluminum clusters produced within the system were predominantly of the alumina type. This is in agreement with previous studies conducted by Ji et al. [25,26]. Figures S1 and S2 in the Supplementary Information illustrate the decomposition process of the HMX@AlH_3_ and CL-20@AlH_3_ NPs at 2400 K, respectively. It was found that the decomposition processes of the HMX@AlH_3_ and CL-20@AlH_3_ NPs were analogous to that of the RDX@AlH_3_ NPs.

### 3.2. Evolution of Total Species

**Figure 3:**
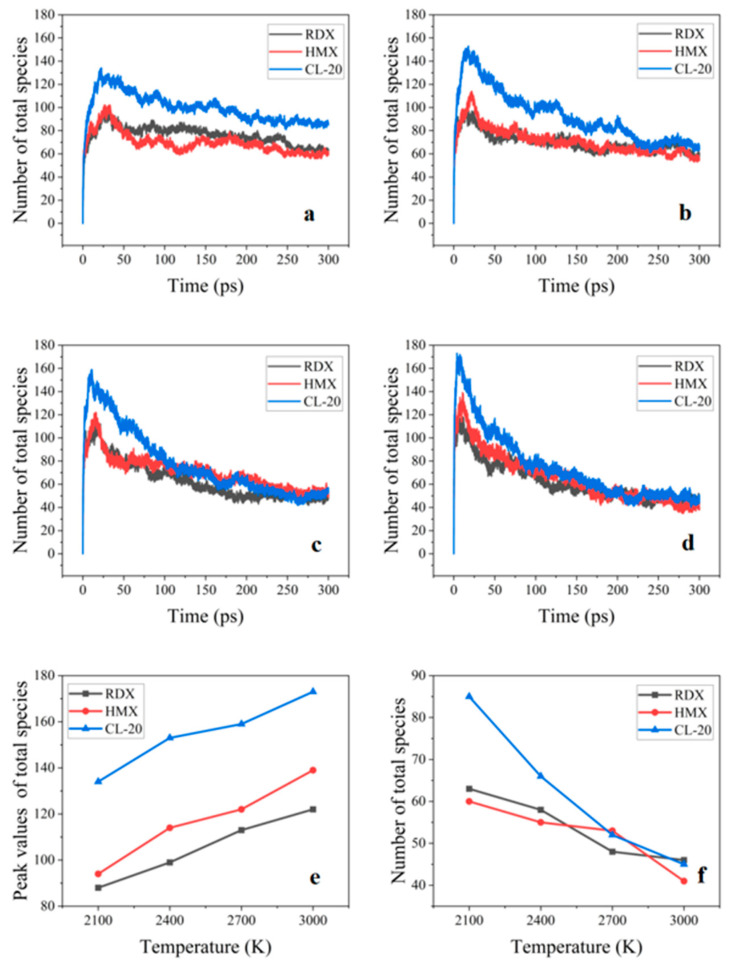
Evolution of the number of the total species in the three AlH_3_-containing explosives with time at (**a**) 2100, (**b**) 2400, (**c**) 2700, and (**d**) 3000 K. Peak values (**e**) and number (**f**) of total species as a function of temperature.

Figure 3a–d illustrate the evolution of the total number of species for the types of AlH_3_-containing explosive NPs at 2100, 2400, 2700, and 3000 K, respectively. As illustrated in Figure 3e, the maximum value of the total number of species for the three systems was observed to increase with the increasing reaction temperature. This suggests that elevated temperatures can accelerate the decomposition of the reactants during the initial decomposition. As the reaction progressed, the total number of species in the three systems gradually approached an equilibrium. At the temperature from 2400 to 3000 K, the number of final species in the three systems exhibited a minimal discrepancy. Nevertheless, at 2100 K, the total number of species in the CL-20@AlH_3_ NP system was markedly higher than those for the RDX and HMX@AlH_3_ NPs. This was due to the fact that the CL-20 molecules are more sensitive to thermal stimuli than the RDX and HMX molecules. Consequently, at lower temperatures, the CL-20 molecules decomposed more completely at a faster rate, resulting in the generation of significantly more molecular fragments than the RDX and HMX@AlH_3_ NPs.

As illustrated in Figure 3f, the total number of species for the three systems exhibited a decline with the increasing reaction temperature in the final stage of the reaction. This was due to the formation of numerous aluminum oxide clusters in the end of the reaction. The high reactivity of the aluminum atoms in the systems allowed them to react with the C, H, O, and N atoms in the RDX, HMX, and CL-20 molecules, consequently forming aluminized clusters and reducing the total number of species.

### 3.3. Gas Products

**Figure 4:**
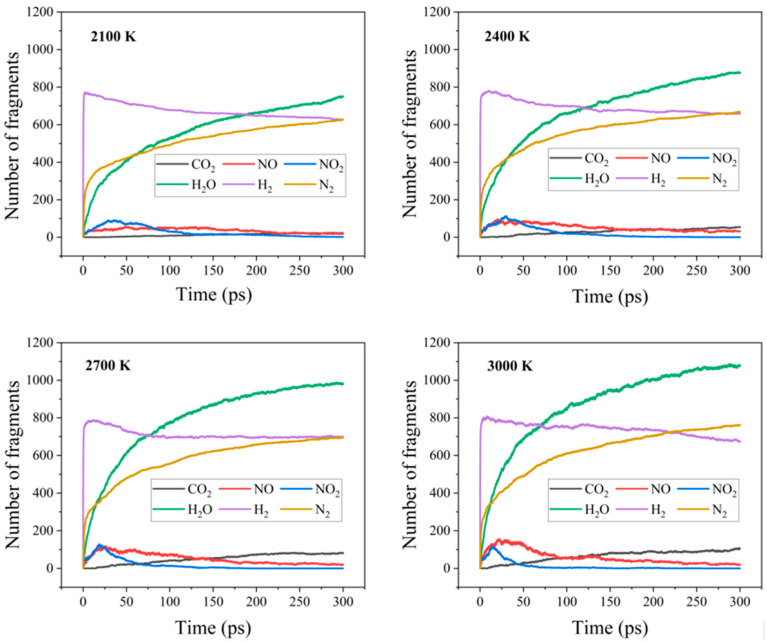
Time evolution of the products in the RDX@AlH_3_ NPs at 2100, 2400, 2700, and 3000 K.

In order to gain detailed insights into the evolution of the gas products during the pyrolysis of the types of AlH_3_-containing explosive NPs, we employed C++ scripts to count the fragments in each system. The selected bond lengths were set to be 1.1 times longer than the standard bond length [8]. Poshevnev et al. [33] determined the gas evolution occurring during the initial pyrolysis of AlH_3_ at temperatures below 100 °C. Figure 4 illustrates the evolution of the gas products in the RDX@AlH_3_ NPs at 2100, 2400, 2700, and 3000 K. As illustrated, during the initial decomposition stage, the number of the intermediate products NO and NO_2_ increased first and then decreased. Furthermore, the formation rate of NO was lower than that of NO_2_, indicating that the formation of NO_2_ was more likely to occur. It was determined that NO_2_ was produced during the decomposition of RDX, typically via the cleavage of the N-NO_2_ bond, as evidenced by the observed trajectory. There are three distinct initial decomposition pathways: (i) N-NO_2_ homolysis; (ii) HONO elimination; (iii) ring-opening reactions. In addition, the incorporation of aluminized hydride shells did not result in any alteration in the initial decomposition mechanism of the three explosives. It could also be observed that the differences between the maxima of the number of NO_2_ and NO fragments decreased with the increasing temperature. At 3000 K, the maximum value of NO exceeded that of NO_2_, indicating that the O-NO bond cleavage was thermodynamically a more favorable path. Furthermore, as the reaction progressed, NO underwent further reactions with the Al atoms, resulting in the formation of N_2_. The concentration of NO subsequently reached a peak, then began to decline, and ultimately reached zero.

As illustrated in Figure 4, the increasing rate of H_2_ formation was essentially consistent at different temperatures, with the evolution of the number of H_2_ fragments exhibiting a similar trend. This suggests that the AlH_3_ shell is a self-dehydrogenating substance, almost unaffected by the temperature. In the mid-stage of the decomposition, the elevated temperature had a slight promoting effect on the release of H_2_, due to the acceleration of the decomposition of RDX at high temperatures. From Figure 4, it can be observed that the yields of H_2_O, CO_2_, and N_2_ increased with the rising temperature. The final yields of the four stabilization products were in the order of H_2_O > H_2_, N_2_ > CO_2_ (2100–2700 K). Meanwhile, in the last stage, the yields of H_2_ and N_2_ were approximately equal, and at 3000 K, they increased in the order of H_2_O > N_2_ > H_2_ > CO_2_.

Figures S3 and S4 in the Supplementary Information illustrate the evolution of the number of gas products in the HMX@AlH_3_ and CL-20@AlH_3_ NPs at different temperatures, respectively. The evolution of the number of gas products in the HMX@AlH_3_ NP system was consistent with that of the RDX@AlH_3_ NPs during pyrolysis. However, in the CL-20@AlH_3_ NPs, the number of final products at 2100 K increased in the following order: H_2_O > H_2_ > N_2_ > CO_2_. In the temperature range from 2400 to 3000 K, the yields of the products increased in the order of H_2_O > N_2_ > H_2_ > CO_2_, which differs significantly from the results observed for the RDX and HMX@AlH_3_ NPs. This may be because CL-20 is very sensitive to the temperature and has a high nitrogen content. This might result in a distinct change in the amount of N_2_ produced, as observed in the CL-20@AlH_3_ NPs, in comparison with the RDX and HMX@AlH_3_ NPs. It is worthy to note that the CL-20 molecule has the highest nitrogen content, resulting in the highest yield of N_2_ among the three explosives.

### 3.4. Reaction Kinetics

**Figure 5:**
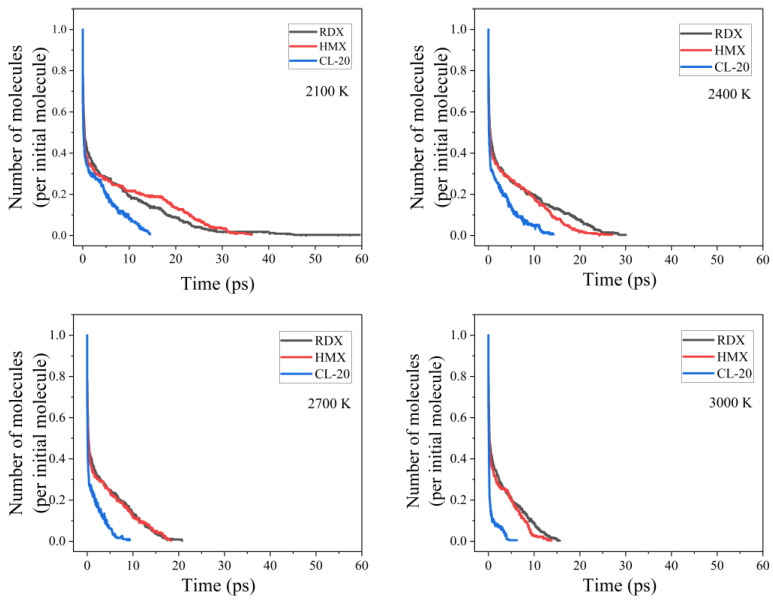
Time evolution of the number of RDX, HMX, and CL-20 molecules at 2100, 2400, 2700, and 3000 K.

Figure 5 illustrates the evolution of the number of RDX, HMX, and CL-20 molecules with time at different temperatures. It was observed that the CL-20 molecules consistently decomposed prior to the RDX and HMX molecules at equivalent temperatures. This was due to the fact that the CL-20 molecules are more sensitive to thermal stimuli and thus decompose more readily. The reaction rate constant *k* for each of the three explosive molecules was obtained by fitting a first-order rate model of the reactants over time, which can be described by the following Equation (1):

N_t_ = N_0_ exp[−*k*(t − t_0_)] (1)

where *k* is the reaction rate constant, t_0_ is the time at which the initial decomposition occurs, *k* is the initial reaction rate constant, N_0_ is the amount of reactants at the start of the reaction, and N_t_ is the amount of reactants at any time. The thermal decomposition rate constant *k* of the three nanoparticles is presented in Table 2 together with their complete decomposition times.

As can be observed from Table 2, the rate constant increased in line with the increasing temperature. This suggests that elevated temperatures facilitated the decomposition of the explosive molecules, thereby reducing the overall decomposition time. Furthermore, the RDX molecule required the longest time to complete the decomposition, indicating that it is the most resistant to thermal stimuli.

### 3.5. Cluster Analysis

**Figure 6:**
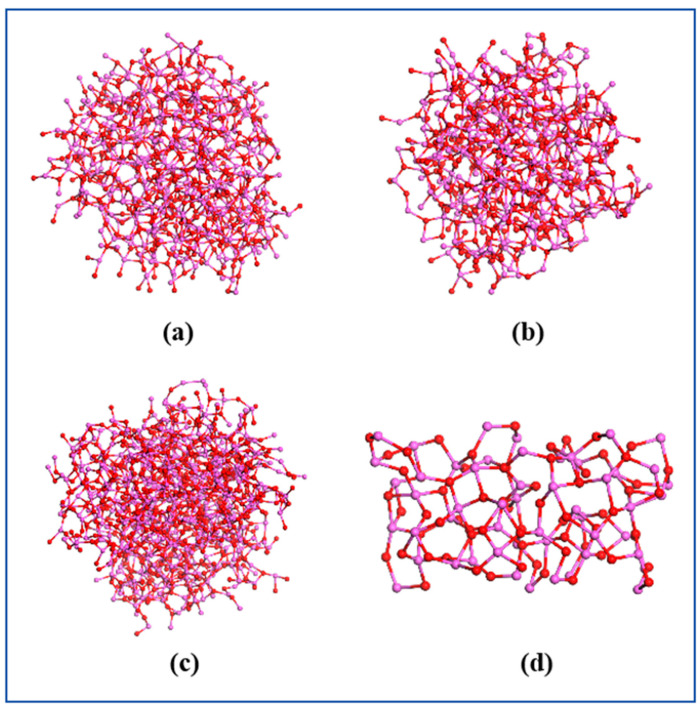
Al_m_O_n_ clusters in RDX @AlH_3_ (**a**), HMX@AlH_3_ (**b**), and CL-20@AlH_3_ (**c**) NPs, and their basic unit (**d**).

The pyrolysis of the three types of AlH_3_-containing explosive NPs at elevated temperatures resulted in the production of not only many gaseous products, but also a solid mixture of aluminum and carbon clusters. It was demonstrated that carbon clusters can be produced during the detonation of explosives [34,35]. The RMD has been effectively employed to investigate the formation of carbon clusters during the detonation of explosives [36]. Figure 6 illustrates the structural composition of the Al_m_O_n_ clusters and their fundamental units in the three types of AlH_3_-containing explosive NPs at 2400 K. As illustrated, the clusters in the three types of nanoparticles exhibited a spherical structure and were stacked by similar primitives. The fundamental unit was a zeolite-like three-dimensional structure, as depicted in Figure 6d.

**Figure 7:**
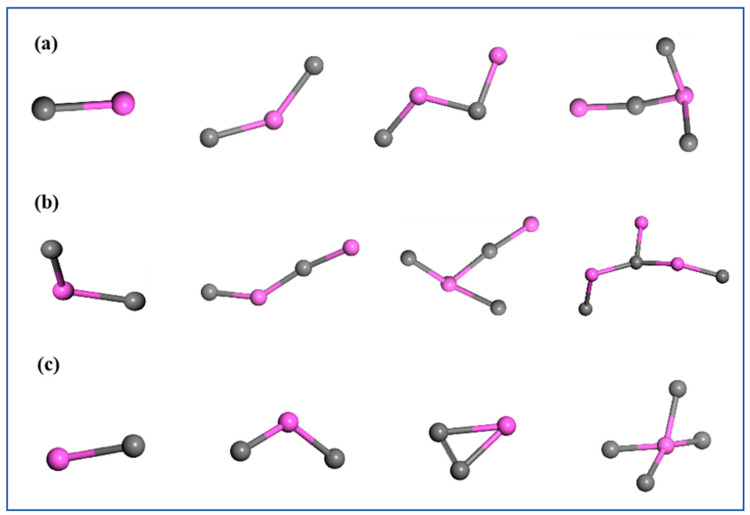
Al_m_C_n_ clusters in the RDX @AlH_3_ (**a**), HMX@AlH_3_ (**b**), and CL-20@AlH_3_ (**c**) NPs.

Figure 7 illustrates the structure of the Al_m_C_n_ clusters in the three types of AlH_3_-containing explosive NPs. It was demonstrated that the Al_m_C_n_ clusters exist as small molecules and fragments in the RDX@AlH_3_, HMX@AlH_3_, and CL-20@AlH_3_ NPs [22]. Furthermore, Ji et al. demonstrated that the Al_m_C_n_ and Al_m_O_n_ clusters are the primary constituents of solid-state aluminized clusters, with Al_m_N_n_ clusters distributed among them. This finding is consistent with our RMD simulation results. In addition, the simulated results indicated that there were no Al_m_H_n_ clusters within the aluminized clusters. This was attributed to the inherent instability of the Al-O bond at elevated temperatures, which renders it susceptible to fracture.

## 4. Conclusions

In this work, the RMD was employed to investigate the thermal decomposition of three types of AlH_3_-containing explosive NPs at high temperatures (2100, 2400, 2700, and 3000 K). It was demonstrated that the CL-20 molecules in the CL-20@AlH_3_ NPs decomposed at a faster rate than the other molecules in the other NPs. An increase in the temperature resulted in a faster initial decomposition rate of the explosive molecules. The evolution of gaseous products during the decomposition of the HMX@AlH_3_ NPs and CL-20@AlH_3_ NPs followed a similar trend to that observed for RDX. The primary initial decomposition mechanisms of the three explosives were as follows: (i) N-NO_2_ homolysis; (ii) HONO elimination; (iii) ring-opening reactions. The variation in the yield of final products with the temperature in the CL-20@AlH_3_ NP system was different from those in the RDX@AlH_3_ and HMX@AlH_3_ NPs. There was a large number of solid aluminized clusters produced in the decomposition, mainly Al_m_O_n_ and AlmCn clusters, together with Al_m_N_n_ clusters dispersed in the Al_m_O_n_ clusters. The Al_m_O_n_ clusters showed a sphere-like structure and a packing basic unit, similar to zeolite with three-dimensional structure. In contrast, the Al_m_C_n_ clusters existed in the form of small molecules and fragments. Our results may provide a theoretical basis for understanding the reaction mechanisms of metal hydride composite explosives and for the design of high-energy aluminized explosives.

## Figures and Tables

**Table 1:** Initial parameters of the RDX@AlH_3_, HMX@AlH_3_, and CL-20@AlH_3_ NPs.

Model	Atoms	Explosive Molecules	No. of AlH_3_	Mass Ratio of AlH_3_
RDX@AlH_3_ NP	9545	332	668	21.4%
HMX@AlH_3_ NP	9772	257	668	20.9%
CL-20@AlH_3_ NP	9488	192	668	19.2%

**Table 2:** Decay time (t, ps) to zero of the reactants and reaction rate constant (*k*, ps^−1^) in the decomposition of the three types of AlH_3_-containing explosive NPs at different temperatures.

	RDX@AlH_3_ NP		HMX@AlH_3_ NP		CL-20@AlH_3_ NP	
	t, ps	*k*, ps^−1^	t, ps	*k*, ps^−1^	t, ps	*k*, ps^−1^
2100 K	59.6	0.257	36.4	0.246	14.5	0.514
2400 K	30.1	0.468	27.1	0.306	14.2	0.848
2700 K	20.8	0.364	18.5	0.404	9.3	1.722
3000 K	15.6	0.282	13.8	0.563	6.2	5.522

## Data Availability

Data are contained within the article and Supplementary Materials.

## Supplementary Materials

The following supporting information can be downloaded at: https://www.mdpi.com/article/10.3390/nano14221859/s1, Figure S1. Snapshots of the morphology the decomposition process of the HMX@AlH_3_ at 2400 K. Figure S2. Snapshots of the morphology the decomposition process of the CL-20@AlH_3_ at 2400 K. Figure S3. Time evolution of the products of the HMX@AlH_3_ NP at 2100, 2400, 2700, and 3000 K. Figure S4. Time evolution of the products of the CL-20@AlH_3_ NP at 2100, 2400, 2700, and 3000 K.
