# Supplementary material for: Thermal Decomposition of Core–Shell-Structured RDX@AlH3, HMX@AlH3, and CL-20@AlH3 Nanoparticles: Reactive Molecular Dynamics Simulations
Source: Nanomaterials (Basel). 2024 Nov 20;14(22):1859. doi: 10.3390/nano14221859 (PMC11597835; doi:10.3390/nano14221859)
Supplement: Supplementary file 1 [file nanomaterials-14-01859-s001.zip › nanomaterials-3301221-supplementary.pdf]

Supplementary Information

Thermal decomposition of core–shell structured RDX@AlH_3, HMX@AlH_3, and CL-20@AlH_3 nanoparticles: Reactive molecular dynamics simulations

Zijian Sun, Lei Yang, Hui Li, Mengyun Mei, Lixin Ye, Jiake Fan and Weihua Zhu*

Institute for Computation in Molecular and Materials Science, School of Chemistry and Chemical Engineering, Nanjing University of Science and Technology, Nanjing 210094, China

* Correspondence: zhuwh@njust.edu.cn

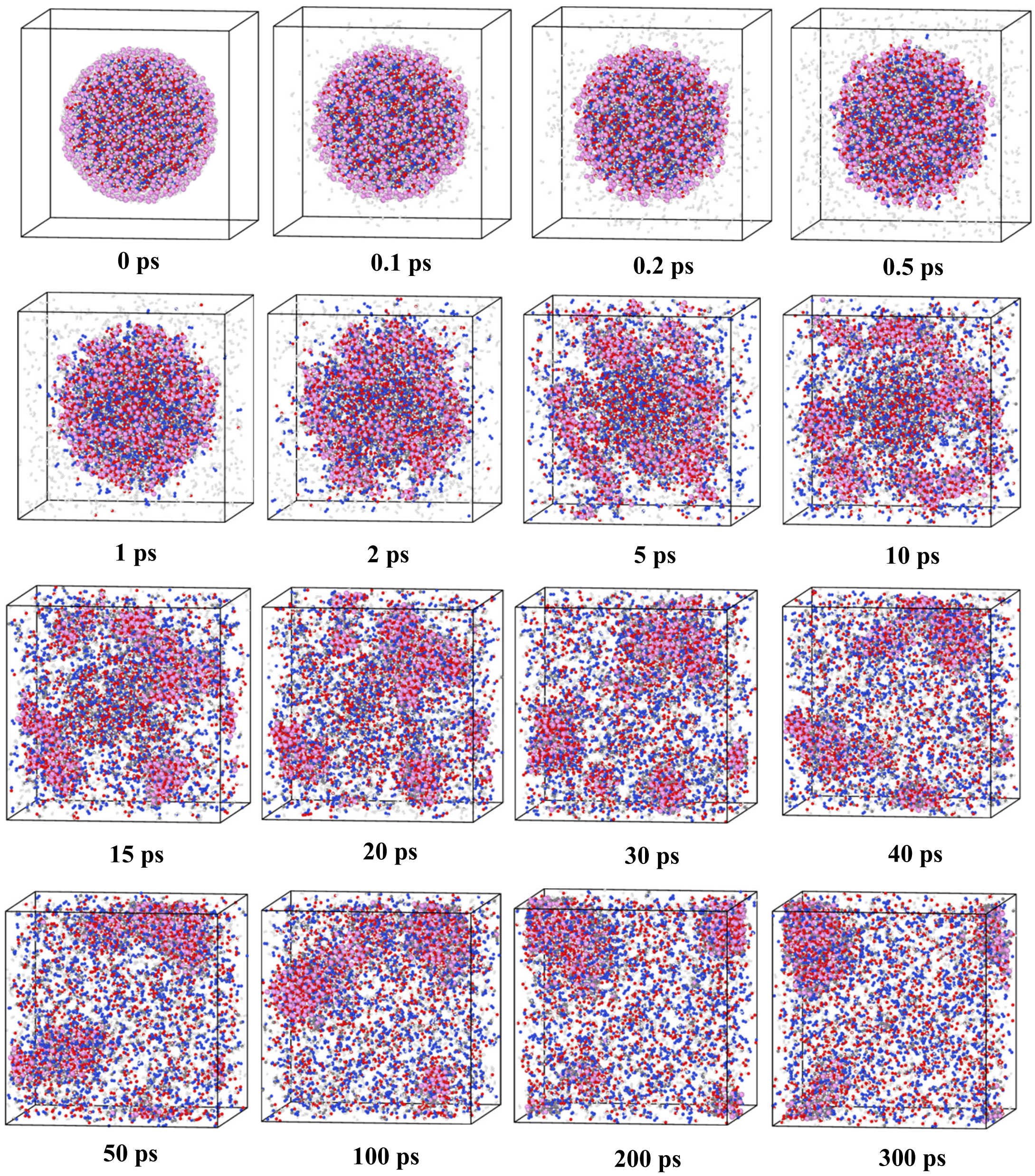

Figure S1. Snapshots of the morphology the decomposition process of the HMX@AlH_3 at 2400 K.

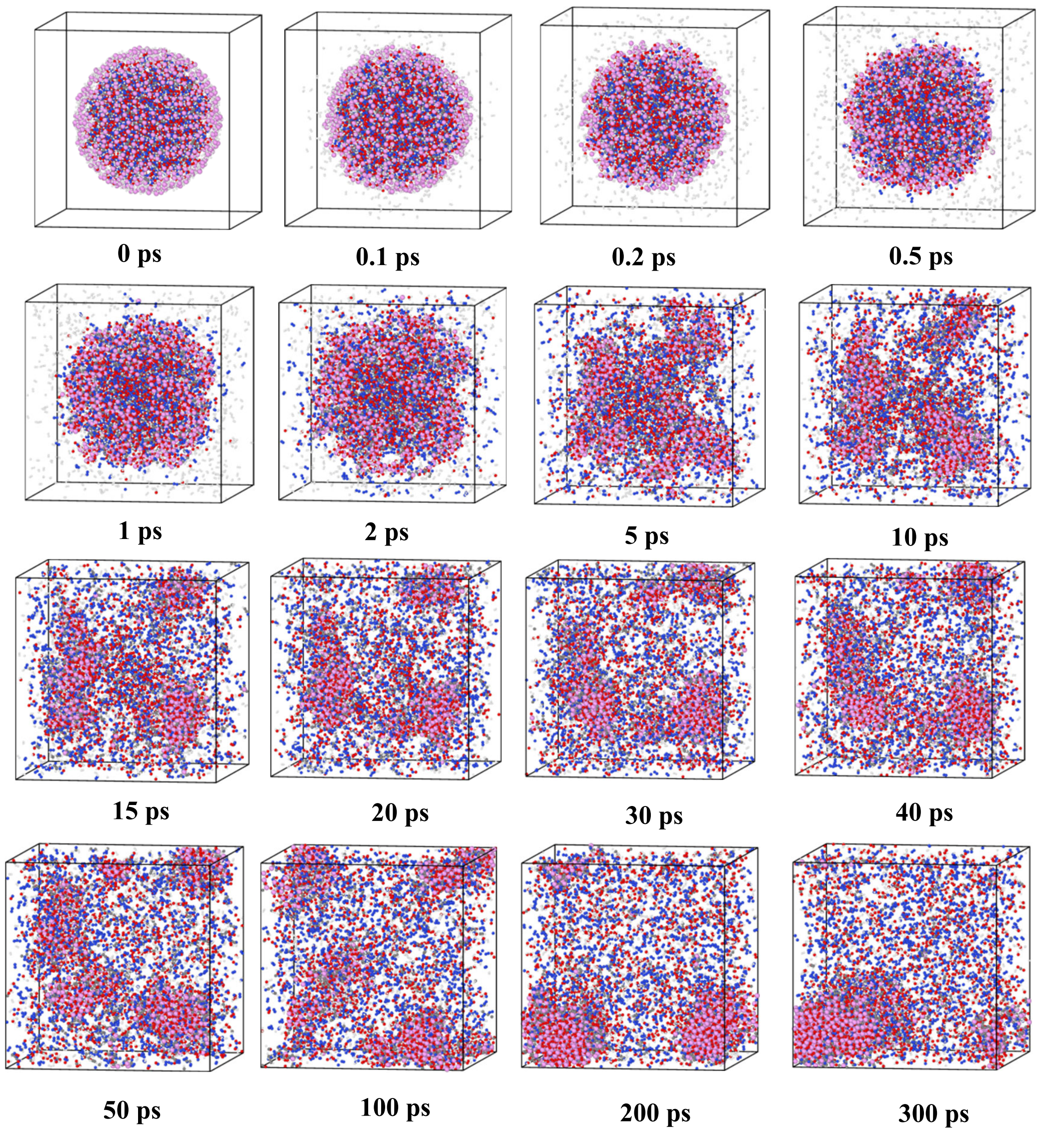

Figure S2. Snapshots of the morphology the decomposition process of the CL-20@AlH_3 at 2400 K.

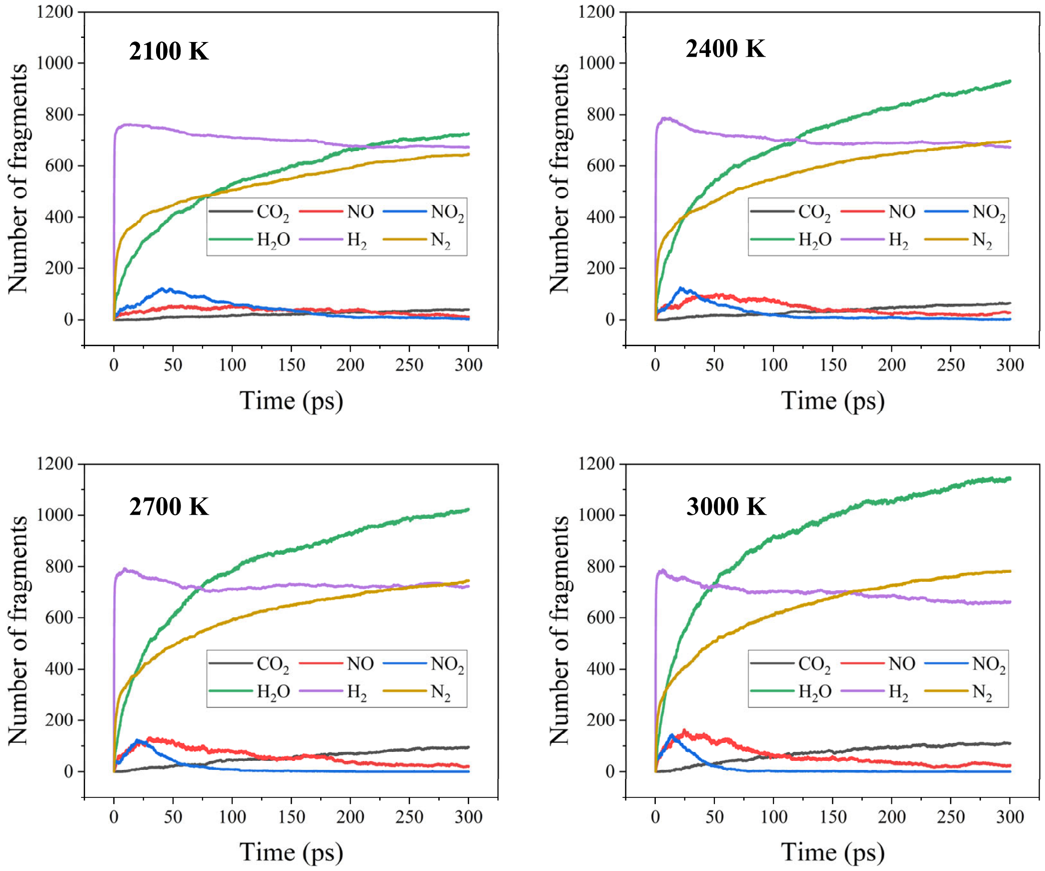

Figure S3. Time evolution of the products of the HMX@AlH_3 NP at 2100, 2400, 2700, and 3000 K.

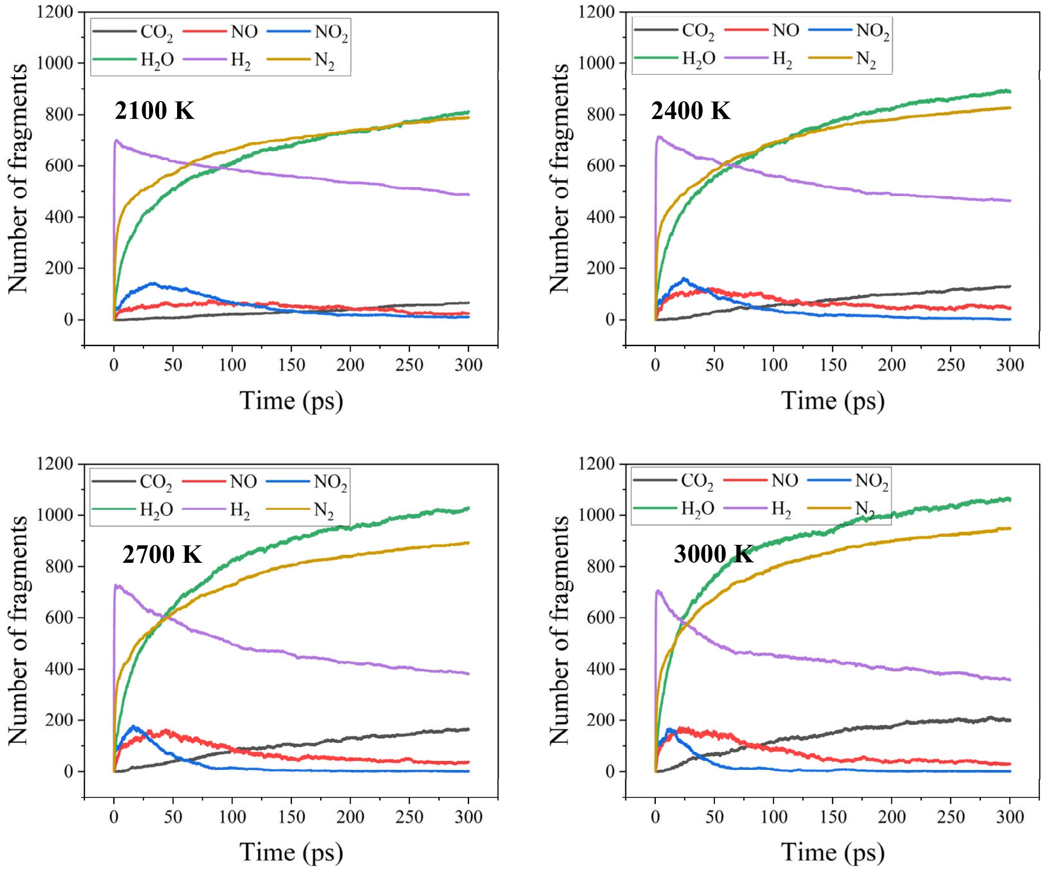

Figure S4. Time evolution of the products of the CL-20@AlH_3 NP at 2100, 2400, 2700, and 3000 K.
